# Fasciolosis in a traveler returning from Burkina Faso

**DOI:** 10.1016/j.fawpar.2026.e00319

**Published:** 2026-02-03

**Authors:** Laurencie Massamba, Jean Testa, Pierre Marty, Jacques Sevestre

**Affiliations:** a Parasitologie-Mycologie, Médecine Tropicale, Faculté de Médecine, Université Côte d'Azur, CHU l'Archet, CS 23079, 06202 Nice, cedex 03, France; b Département de santé publique, Université Nazi Boni, Bobo Dioulasso, Burkina Faso; c URE RETINES, Faculté de Médecine, Université Côte d'Azur, France; d Aix-Marseille Université, SSA, AP-HM, RITMES, Marseille, France; e IHU-Méditerranée Infection, Marseille, France

**Keywords:** Fasciolosis, Burkina Faso, Africa, Distomatosis, Hypereosinophilia

## Abstract

Human distomatoses may be caused by several genera of trematodes, including *Fasciola* spp., responsible for cosmopolitan fasciolosis. Once widespread in Western Europe, the prevalence of these parasitoses has significantly declined in the last decades. The rarity of these infections may result in overlooking such etiologies. Nevertheless, imported cases are still being diagnosed, notably among migrants and travelers returning from endemic areas. Laboratory assays used for confirmation require different techniques, which exhibit various sensitivities and specificities, thus requiring expertise. In this context, diagnosis of fasciolosis can be challenging, particularly in primary care settings. We present a case of hepatic fasciolosis, likely acquired in Burkina Faso, in a traveler for whom several months elapsed before etiological diagnosis was made. Given the important number of patients visiting endemic areas, and recent migratory movements, the incidence of human imported distomatosis may increase in metropolitan France in the near future.

## Highlights

• Human fasciolosis is a zoonotic trematodosis with a global distribution.
• Autochtonous infections are nowadays rare in Europe but travelers may be at risk.
• Clinical suspicion should arise in eosinophilia, prompting appropriate testing.
• Treatment relies on administration of triclabendazole in two separate doses.

## 1 Introduction

Human distomatoses are parasitic zoonoses which may be caused by various genera of trematodes (Keiser and Utzinger, 2009). Given the diversity of genera involved in human medicine, and different anatomical regions possibly involved, the resulting infestations may cause a variety of clinical pictures (Keiser and Utzinger, 2009). In humans, who are accidental hosts, most infestations are linked to the consumption of raw plants grown in submerged conditions and involve the hepatobiliary tree (Keiser and Utzinger, 2009). Distomatoses notably encompass fasciolosis, for which the causative agents belong to the *Fasciola* spp. genus, including *Fasciola hepatica* and *F. gigantica*. *F. hepatica* is globally distributed, with an established presence on all five continents, whereas *F. gigantica* distribution is restricted to tropical regions of Africa and Asia (Keiser and Utzinger, 2009).

In Western Europe, the incidence of autochthonous hepatic fasciolosis caused by *F. hepatica* significantly decreased in recent decades (Mas-Coma, 2005). Nevertheless, it is currently estimated that some 180 million people worldwide live in conditions that place them at risk for *F. hepatica* fasciolosis, with an estimation of 2 up to 17 million infected patients in more than 50 countries (Mas-Coma et al., 2009).

Within the *Fasciola* genus, *F. gigantica* is endemic in tropical countries, including Southeast Asia and sub-Saharan Africa. Notwithstanding its geographical distribution, this species undergoes a similar parasitic cycle in its definitive hosts, causing disease courses similar to *F. hepatica* (Keiser and Utzinger, 2009). Although prevalence data for *F. gigantica* infection is scarce, it may be reasonably assumed that an important number of people are at risk of being infested by this agent. Thus, fasciolosis continues to represent a major health issue worldwide, categorized by the World Health Organization as a Neglected Tropical Disease (Foodborne trematode infections, 2025).

Given the rarity of autochthonous and travel-associated fasciolosis in contemporary metropolitan France, such diagnosis may easily be overlooked in primary care settings (Ashrafi et al., 2014). Moreover, ectopic forms of the disease may complicate clinical assessment (Mowlavi et al., 2023). Furthermore, the low sensitivity of parasitological stool examination in human infestations further hampers definitive diagnosis (Arjona et al., 1995). Nevertheless, precise patient interrogation and the presence of specific paraclinical findings may prompt a diagnosis of fasciolosis in patients for whom digestive parasitosis is suspected (Ashrafi et al., 2014).

We report a case of human fasciolosis in a patient who had travelled to Burkina Faso, and who sought care for several months before a diagnosis was made.

## 2 Case report

A 54-year-old male patient presented in August 2018 for evaluation of a possible digestive parasitosis, with digestive and cutaneous symptoms accompanied by peripheral hypereosinophilia, following recent travel to Burkina Faso. During clinical interview, no significant medical history was reported. No allergies were reported. No medication had been introduced in the months preceding symptom onset. The patient's recent travel history included multiple visits to Burkina Faso for participation in agricultural development projects. The last trip took place in January 2017 and occurred in the rural areas of Koudougou and Toma.

Upon questioning, the patient reported consumption of raw vegetables grown underwater during his last stay, including lettuce. No baths, voluntary or accidental, were reported. Following his return in March 2017, he noted the appearance of fluctuating fever, associated with digestive signs (including diarrhea and rectal syndrome) and pain in the right upper quadrant. These manifestations were associated with intense pruritus and subsequent development of a subcutaneous nodular lesion located in the right upper quadrant. The intensity of the signs was reported to fluctuate over the past months.

**Fig. 1:**
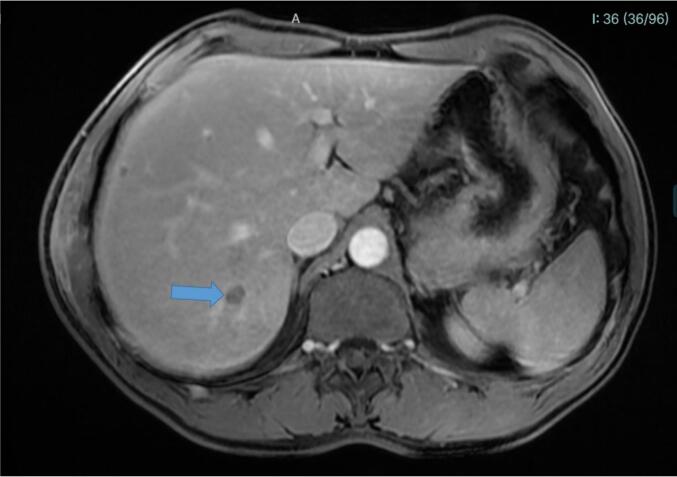
Gadolinium-injected Liver Abdominal Magnetic Resonance Imaging (tardive Lava-Flex T1 sequence) in a patient diagnosed with fasciolosis. The figure is notable for the presence of a non-enhancing lesion consistent with parenchymal abscess (arrow).

**Fig. 2:**
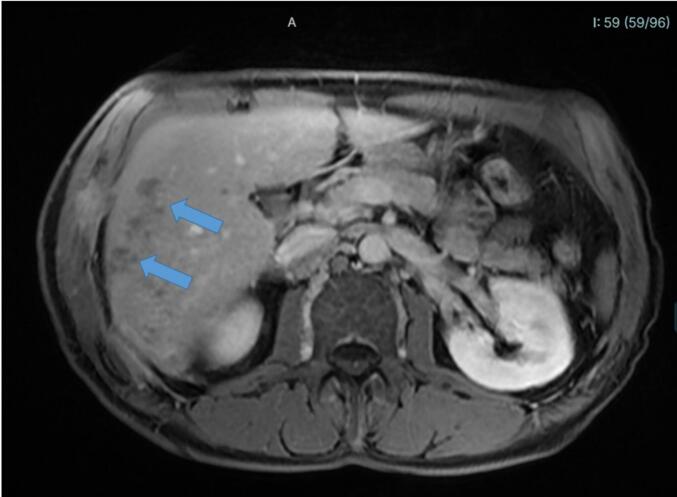
Liver Abdominal Magnetic Resonance Imaging (Lava-Flex T1 sequence) in a patient diagnosed with fasciolosis. The figure is notable for the presence of tunnel-like lesions (arrows), which are typically associated with hepatic fasciolosis.

At medical assessment in July 2018, laboratory work-up revealed a modest elevation of C-reactive protein (8.8 mg/L; normal <5 mg/L) and marked hypereosinophilia (4712 eosinophils/mm^3^; normal <500/mm^3^). All other parameters were within normal range. Several parasitological examinations, including wet mount and Bailenger's sedimentation method, yielded negative results. Radiological investigations, including thoracoabdominal ultrasound, revealed a heterogeneous patch in the hepatic dome and an atypical nodular lesion measuring 31 mm within the perihepatic intercostal muscles. Thoraco-abdominal computed tomography scanning (CTS) revealed a 7-mm nodule in the lower lobe of the right lung, as well as heterogeneous liver parenchymal lesions associated with subhepatic effusion. Hepatic nuclear magnetic resonance imaging (MRI) was also performed and revealed the presence of micronodular hepatic lesions associated with several tunnel-like tracts (Fig. 1, Fig. 2).

In light of the above findings, the patient was referred to parasitology specialists at the Centre Hospitalier Universitaire de Nice (Nice, France), in mid-August 2018. Serological testing was performed using the *Fasciola* IgG Western Blot (LDBio Diagnostics, Lyon, France), which revealed the presence of 8–9 kDa, 28 kDa and 42 kDa bands. In this context, a diagnosis of hepatic fasciolosis was made, and treatment with triclabendazole was introduced, with a single oral dose of 750 mg. A few days later, the patient self-medicated with the same dose of triclabendazole, which he had obtained on his own. At a follow-up visit one month later, no active symptoms were reported. Laboratory workup performed in September 2018 showed a decrease in eosinophilic count to 700/mm^3^. Other parameters were within the normal range. By November 2018, the blood eosinophil count had decreased to 500/mm^3^, corresponding to normalization. On the last follow-up visit in October 2019, fourteen months after treatment, no symptoms were reported. Clinical examination did not retrieve any abnormality. Eosinophil count was within the normal range (400/ mm^3^). A follow-up MRI exam showed stability of nodular hepatic lesions compared to previous examinations. Thus, the patient was considered cured.

## 3 Discussion

We report a case of hepatic fasciolosis diagnosed in a patient with relevant exposure through multiple travels to tropical Africa.

Human fasciolosis is a neglected disease in Africa. Dermauw et al. (2021) state that cases were reported in only 12 African countries, namely Algeria, Angola, Cape Verde, Egypt, Ethiopia, Ghana, Morocco, Nigeria, Senegal, South-Africa, Tanzania and Tunisia, and that very few of these cases were reported south of Sahara. In our patient's case, case history revealed consumption of salads (e.g. lettuce) and other raw vegetables grown in submersion, near to cattle herds. *Fasciola* flatworms have a parasitic cycle involving different aquatic gastropod species as intermediate hosts (Dermauw et al., 2021). Their metacercarial forms generally attach themselves to submerged plants, thus enabling ingestion by mammals in which the trematode can complete its cycle (Arjona et al., 1995). This developmental cycle is common to both *F. hepatica* and *F. gigantica*, which cause similar clinical pictures in humans (De et al., 2025). However, only *F. gigantica* is endemic to West Africa, as *Galba/Fossaria* vectors of *F. hepatica* have never been reported in this area (Nukeri et al., 2022). Only *Radix natalensis*, the snail vector of *F. gigantica,* has been reported in countries in this geographical area (Mas-Coma et al., 2009). The sole presence of this trematode species had been confirmed previously through extensive phenotypic and molecular studies (Mas-Coma et al., 2009; Periago et al., 2006). A recent study highlighted the presence of *F. gigantica* in over 4.5% of the livers of cattle slaughtered in Bobo-Dioulasso Zooparaz.net - Scientia Parasitologica, 2025). Although infections due to both species have previously been reported in sub-Saharan slaughterhouses, cattle infected by *F. hepatica* likely originated from Europe, where this species is endemic (Mas-Coma et al., 2022).

In human infection, species-level identification of the parasite may rely on the microscopic morphology of eggs and can also be achieved through molecular methods (Cabada and White, 2012). However, morphological identification of eggs is complex, owing to substantial overlap in dimensions and morphometric characteristics between *F. hepatica* and *F. gigantica* (Cabada and White, 2012; Valero et al., 2009). Moreover, the possible existence of hybrid forms could further hamper this method (Nukeri et al., 2022). In our patient, stool examination did not retrieve parasite eggs, as frequently observed in human infestations. Indeed, intermittent shedding, low worm burden, and ectopic biliary localizations are also known to hamper microscopic detection of eggs, particularly in *F. gigantica* fasciolosis (Valero et al., 2009). In the absence of stool qPCR testing, no further investigations could allow identification of the species involved in our patient. Nevertheless, taking into account the *Fasciola* species distribution, our patient was most likely infected with *F. gigantica* (Mas-Coma et al., 2009; Periago et al., 2006).

Parasitological diagnosis of fasciolosis can be challenging due to several factors. In non-hyperendemic regions, the low incidence of this parasitosis may result in this diagnosis being overlooked, even in the presence of hypereosinophilia. Indeed, the majority of hypereosinophilias assessed in metropolitan France are the result of immunoallergic reactions (Groh et al., 2023). Furthermore, the frequency of hepatic fasciolosis in France has declined significantly over the last century (Mas-Coma, 2020). These aspects may explain the medical wandering experienced by our patient. In addition, parasitological tests used to diagnose fasciolosis display different sensitivities (Mas-Coma et al., 2014). The detection of eggs in stools is often poorly sensitive due to the reasons stated above (Keiser and Utzinger, 2009). Repeated stool examination is often necessary to observe eggs. Nevertheless, in the acute phase of the disease stool parasitology is of limited diagnostic value, which might have been a limiting factor in our patient's case. Furthermore, in cases of ectopic fasciolosis, stool parasitology may remain negative since the eggs are not excreted in the bile ducts (Mas-Coma et al., 2014; Taghipour et al., 2019).

Although indirect diagnostic techniques, serological tests may compensate for the low sensitivity of stool examination in the diagnosis of hepatic fasciolosis. (Mas-Coma et al., 2014; Mirzadeh et al., 2021). In this parasitosis, antibody production is preceded by the onset of hypereosinophilia, which coincides with the parasite tissue invasion phase. Various serological techniques can detect antibodies as early as four to seven weeks before excretion of eggs in stool (Keiser and Utzinger, 2009). This method is particularly useful in ectopic fasciolosis, where eggs may not be excreted in the bile ducts (Mowlavi et al., 2023). Imaging studies, whether ultrasound, CTS or MRI, can also contribute to the diagnosis of fasciolosis (Dusak et al., 2012; Preza et al., 2019). The most frequently reported radiological signs are confluent hypodense nodular formations, enhanced by the addition of intravenous contrast medium, and branching serpiginous tunnel-like lesions, consequence of larval migration (Dusak et al., 2012; Preza et al., 2019). Perihepatic effusions may be associated with hepatic distomatosis (Preza et al., 2019). Different radiological aspects may be observed depending on the stage of the disease (i.e. parenchymal or biliary phase). In the case of ectopic localization, abdominal wall involvement has also been reported (Taghipour et al., 2019). In our patient's case, direct observation of the parasite could not be achieved. Such diagnosis is based on the observation of eggs, or through molecular or antigenic detection in the stool. It has been assessed that fasciolosis diagnosis should rely on combination of different diagnostic techniques, involving at least blood and stool sampling (Mas-Coma et al., 2014). It can also be obtained by histopathological analysis of a biopsy specimen. However, this invasive procedure may be iatrogenic. In our patient's case, all the anamnestic (consumption of raw vegetables in an endemic area), clinical (clinical signs of larval invasion), biological (hypereosinophilia and positive western blot testing for specific antibodies) and radiological findings (presence of confluent nodular hepatic lesions) helped ascertain the diagnosis. In case of positive diagnosis, treatment consists of oral triclabendazole given in two doses, separated by 12 h (De et al., 2025). Moreover, clinical, biological and radiological improvement following specific treatment confirms the diagnosis of imported hepatic fasciolosis in a patient cured with two courses of triclabendazole.

## 4 Conclusion

We present a case of imported hepatic fasciolosis, most likely due to *F. gigantica,* with ectopic migration, in a patient who had spent time in an endemic zone and was exposed to the consumption of lettuce. This is probably the first case reported in Burkina Faso. Although the incidence of this parasitosis has declined significantly in Western Europe, imported cases persist, particularly in metropolitan France. Clinicians should bear in mind the possibility of an uncommon digestive parasitosis in patients presenting with hypereosinophilia on return from travel. In such cases, clinicians should call on experts in parasitology to guide the examination strategy, ensuring that diagnostic tests are selected and interpreted in the most appropriate context.

## CRediT authorship contribution statement

**Laurencie Massamba:** Formal analysis, Data curation, Writing – original draft. **Jean Testa:** Validation, Supervision, Writing – review & editing. **Pierre Marty:** Validation, Supervision, Formal analysis, Data curation, Writing – review & editing. **Jacques Sevestre:** Formal analysis, Writing – review & editing, Writing – original draft.

## Consent to publish

Written informed consent was obtained from the patient for publication of the clinical case.

## Funding

Not applicable.

## Declaration of competing interest

The authors declare that they have no known competing financial interests or personal relationships that could have appeared to influence the work reported in this paper.

## Data Availability

Data are available upon reasonable request.
